# Sentinel hospital-based surveillance for norovirus infection in children with gastroenteritis between 2015 and 2016 in Italy

**DOI:** 10.1371/journal.pone.0208184

**Published:** 2018-12-14

**Authors:** Simona De Grazia, Gianvito Lanave, Giovanni Maurizio Giammanco, Maria Cristina Medici, Flora De Conto, Fabio Tummolo, Adriana Calderaro, Floriana Bonura, Noemi Urone, Anna Morea, Daniela Loconsole, Cristiana Catella, Mariarosaria Marinaro, Antonio Parisi, Vito Martella, Maria Chironna

**Affiliations:** 1 Dipartimento di Scienze per la Promozione della Salute e Materno Infantile “G. D’Alessandro”, Università di Palermo, Palermo, Italy; 2 Dipartimento di Medicina Veterinaria, Università Aldo Moro di Bari, Valenzano, Italy; 3 Dipartimento di Medicina e Chirurgia, Università degli Studi di Parma, Parma, Italy; 4 Dipartimento di Scienze Biomediche e Oncologia Umana, Università Aldo Moro di Bari, Bari, Italy; 5 Dipartimento di Malattie Infettive, Parassitarie ed Immunomediate, Istituto Superiore di Sanità, Rome, Italy; 6 Istituto Zooprofilattico Sperimentale di Puglia e Basilicata, Foggia, Italy; University of Liverpool, UNITED KINGDOM

## Abstract

Noroviruses are one of the leading causes of gastro-enteric diseases worldwide in all age groups. Novel epidemic noroviruses with GII.P16 polymerase and GII.2 or GII.4 capsid type have emerged worldwide in late 2015 and in 2016. We performed a molecular epidemiological study of the noroviruses circulating in Italy to investigate the emergence of new norovirus strains. Sentinel hospital-based surveillance, in three different Italian regions, revealed increased prevalence of norovirus infection in children (<15 years) in 2016 (14.4% versus 9.8% in 2015) and the emergence of GII.P16 strains in late 2016, which accounted for 23.0% of norovirus infections. The majority of the strains with a GII.P16 polymerase showed a GII.2 capsid genotype (79.5%). Also, a marked circulation of strains with a GII.17 capsid (14.0%) was observed, chiefly in early 2016. The emergence and global spread of non-GII.4 noroviruses pose challenges for the development of vaccine strategies.

## Introduction

Noroviruses (NoVs) are a major cause of acute gastroenteritis in children and adults worldwide [1]. NoVs are classified into at least seven genogroups (GI to GVII) with genogroup GI, II and IV causing diseases in humans [2,3]. Human NoVs have been divided into several genotypes based on the sequences of polymerase (pol) and major viral capsid protein (cap), coded by the ORF1 and ORF2, respectively. Polymerase genotypes are indicated by a P preceding the assigned classification number (e.g. GII.P4) [4]. In previous decades, symptomatic NoV infection in humans has mostly been sustained by a single cap genotype, GII.4, responsible for about 70% of outbreaks [4,5]. GII.4 NoVs quickly evolve into variants through accumulations of point mutations and recombination involving both pol and cap genes [6,7].

In recent years, novel non-GII.4 NoV strains have been observed as predominant genotypes in children hospitalized with acute gastroenteritis in several countries [8,9]. During the winter season 2014/15 a novel GII.P17_GII.17 NoV strain, Kawasaki 2014, emerged in several Asian countries, replacing the pandemic strain GII.4 Sydney 2012, and subsequently spreading to non-Asiatic countries [8,10]. In late 2015 and in 2016, recombinant GII.P16_GII.2 NoV strains emerged in Asian [11] and European countries [12,13], whilst GII.P16_GII.4 viruses were predominant in the USA [9].

The novel recombinant GII.P16 viruses possess peculiar amino acid substitutions in the ORF1 protein, close to positions known to influence polymerase function and virus transmission, likely accounting for the epidemic potential of these viruses [14, 15].

From 2012 the Italian Study Group for Enteric Viruses (ISGEV; http://isgev.net) conducts hospital-based surveillance on enteric viruses in Parma (Northern Italy), Bari (Southern Italy) and Palermo (Sicily) [16–18]. In late 2016, ISGEV observed the emergence in Italy of recombinant NoV strains GII.P16_GII.4 and GII.P16_GII.2. To investigate the features of the new NoV variants emerging in Italy, we performed multi-target analysis in the diagnostic region A of pol and C of cap genes for the NoV strains detected in Italy during 2015–2016.

## Materials and methods

### Samples collection

During two consecutive years, 2015 and 2016, of surveillance conducted in Parma, Bari and Palermo, a total of 2184 and 2179 stool samples, respectively, were collected from pediatric patients (<15 years old) hospitalized with acute gastroenteritis. The three hospitals serve a cumulate pediatric population of 421,688 individuals (i.e. 5.1% of the total Italian pediatric population as of November 21^st^, 2017). Collection of faecal samples was part of the general process of diagnosis for acute gastroenteric disease of patients. This study did not require local ethics committee approval since all diagnostic tests were routine. All participants provided written consent to participate. The study was conducted in accordance with Italian and Institutional standards, with the principles set down in the Declaration of Helsinki and its revisions, and with local legislation. Following the guidelines of the Italian Data Protection Act, only limited data elements (including initials, gender and year of birth) were collected from patients, in order to analyze the data anonymously.

### RNA extraction and molecular characterization

**Fig 1:**
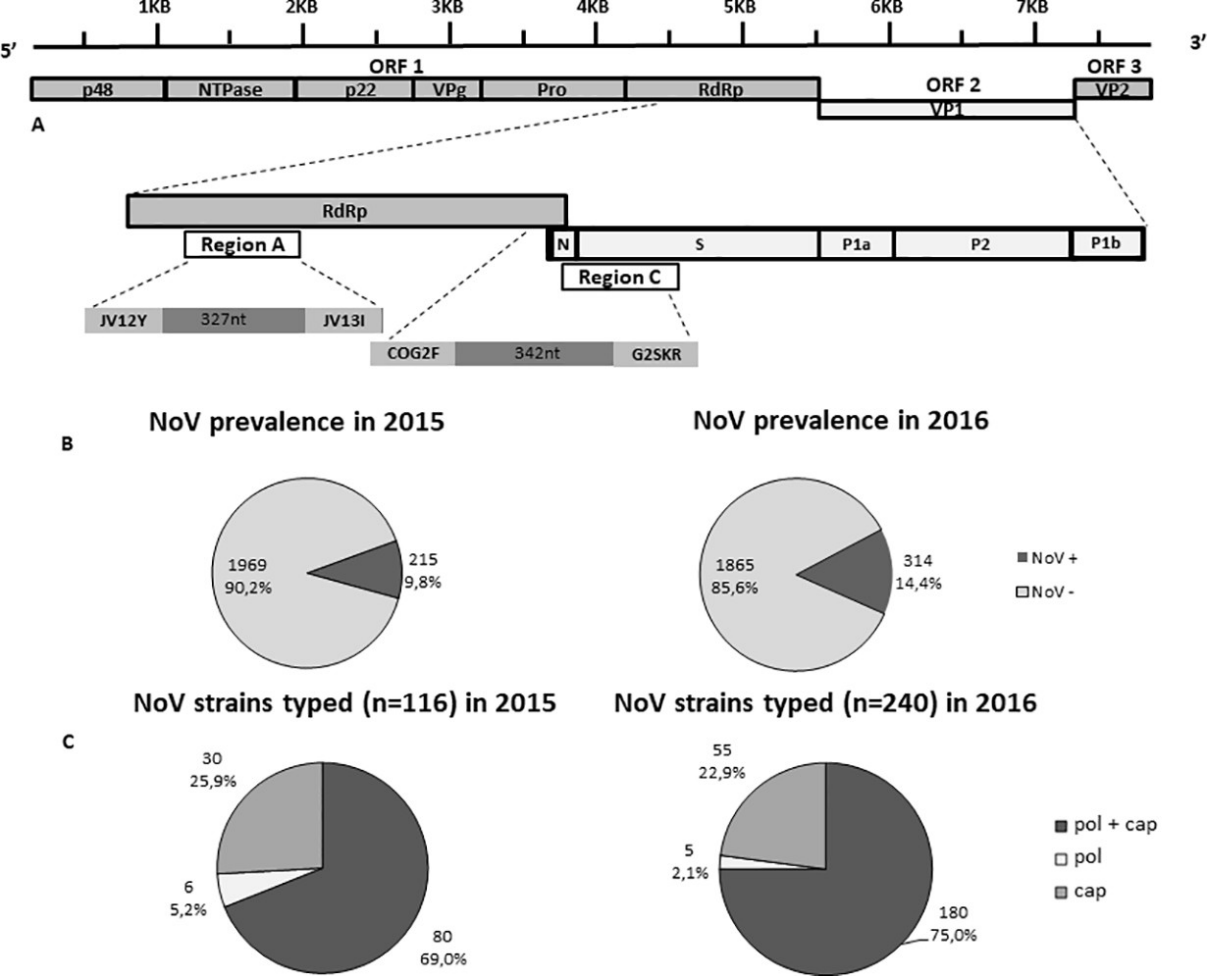
(A) Structure of NoV genome and position of the diagnostic regions A (RNA-dependent RNA polymerase, ORF1) and C (capsid, ORF2). (B) Prevalence of NoVs in 2015 and 2016. (C) Outline of the NoV genotyping data for the strains with complete and partial characterization in regions A and C in the years 2015 and 2016.

Viral RNA was extracted from 140 μl of a 10% stool suspension with a QIAamp Viral RNA Mini Kit (QIAGEN, Hilden, Germany) according to the manufacturer’s instructions. A real-time reverse transcription (RT)-PCR assay, able to differentiate between GI and GII NoV-positive samples, was used to detect NoV RNA [19]. All NoV-positive specimens were genotyped by sequence analysis of the diagnostic region A (ORF1, coding for pol: 327 nt, at positions 4538–4865 relative to U07611 reference sequence) and C (ORF1/ORF2 junction, coding for cap: 342 nt, at positions 5307–5649 relative to U07611 reference sequence), using primers JV12Y/JV13I and COG2F/G2SKR, respectively [19,20] (Fig 1A).

### Sequence analysis

PCR amplicons were purified by commercial columns (CleanSweep PCR Purification, Thermo Fisher Scientific, Vilnius, Lithuania) and sequenced by Sanger method. The nucleotide sequences were edited using Geneious v9.0 software package [21]. The genotypes were determined by using the Norovirus Typing Tool (http://www.rivm.nl/mpf/norovirus/typingtool) and the sequences were deposited in the Noronet-RIVM database [22].

### Statistical analysis

The association between age and NoV genotypes was evaluated statistically by a two-tailed Mann-Whitney U test, using a web-based software program (R version 3.3.0) (http://www.r-project.org). Two-tailed p-values less than 0.05 were considered to be statistically significant. Chi-square analysis was applied to compare the prevalence of NoV infection in 2015–2016.

## Results

ISGEV sentinel hospital-based surveillance for NoV in pediatric patients monitored a nearly 50% increase in NoV prevalence in Italy in 2016 with respect to 2015. NoV prevalence reached 14.4% [95% CI: 13–16] (314/2179) in 2016 vs 9.8% [95% CI: 8.6–11.2] (215/2184) in 2015 (p<0.01) (Fig 1B). In particular, in 2016 NoV prevalence was 22.5% (101/449) in Palermo, 9.1% (74/816) in Parma and 15.2% (139/914) in Bari. In 2015 the prevalence was 16.3% (79/483) in Palermo, 13.9% (83/593) in Parma and 4.8% (53/1108) in Bari.

### Norovirus genotypes circulation

A subset of the 2015 and 2016 NoV-positive samples, 53.9% (116/215) in 2015 and 76.4% (240/314) in 2016, was characterized by sequence analysis of pol and cap genes (Fig 1A) using the Noronet automated genotyping tool. The NoV sequences were deposited in the Noronet database (https://www.rivm.nl/en/noronet/databases). In 2015, complete genotyping (pol and cap types) was obtained in 80 of 116 (69.0%) NoV-positive samples subjected to genotyping, whilst 36/116 (31.0%) were partially typed, either in pol (6/116, 5.2%) or cap (30/116, 25.8%) genes (Fig 1C). The results of cap and pol genotyping of the Italian NoV strains detected in 2015 are reported in Table 1. In 2015, the GII.4 cap genotype accounted for 60.9% (67/110) of the ORF2-typed strains, whilst non-GII.4 accounted for 39.1% (43/110). The predominant pol types were GII.P4 (39.5%, 34/86) and GII.Pe (31.4% 27/86), followed by GII.P7 (10.5%, 9/86) and GII.P21 (7.0%, 6/86). Out of 80 fully-typed strains, 25 (31.2%) showed the predominant cap type GII.4 associated with a GII.Pe pol type (variant Sydney 2012) and 32 (40.0%) with the GII.P4 pol type, derived from the GII.4 variant New Orleans 2009 (Table 1).

**Table 1:** Distribution of pol and cap genotypes in 116 NoV strains typed in 2015. In grey tone the relevant pol/cap genotypes.

	GII.1	GII.2	GII.3	GII.4	GII.6	GII.7	GII.13	GII.14	GII.17	GII.21	GI.1	GI.3	NT	TOT
GII.P2		3		1										4
GII.P4				32									2	34
GII.P7					7			1					1	9
GII.P16							1							1
GII.P17									2					2
GII.P21			3				1			1			1	6
GII.Pe				25									2	27
GII.Pg	3													3
GI.P1														
GI.P3														
NT	3	4	1	9	4	1	2		2	1	2	1		30
TOT	6	7	4	67	11	1	4	1	4	2	2	1	6	116

In 2016, complete genotyping was obtained for 180 of 240 NoV-positive samples (75.0%), whilst 60 (25.0%) were partially typed, either in pol (5/240, 2.0%) or cap (55/240, 22.9%) (Fig 1C). The genotypes of partial and fully typed (pol and cap sequence) NoV strains detected in 2016 are shown in Table 2. In 2016, the GII.4 cap genotype accounted for 40.0% (94/235) of the ORF2- typed strains, whilst non-GII.4 accounted for 60.0% (141/235). The most common non-GII.4 cap types were GII.2 (33.6%, 79/235) and GII.17 (14.0%, 33/235). The predominant pol types were GII.P16 (23.8%, 44/185), GII.Pe (21.1% 39/185), GII.P4 (18.9%, 35/185), GII.P17 (14.1%, 26/185) and GII.P2 (10.3%, 19/185). Out of 180 fully typed strains, the GII.4 cap type was associated with a GII.Pe pol type (variant Sydney 2012) in 39 (21.6%) strains, with the GII.P4 pol type in 33 (18.3%) strains and with the GII.P16 pol type in 7 (3.8%) strains. A total of 35 (19.4%) strains were characterized as GII.P16_GII.2 whilst 18 (10%) were characterized as GII.P2_GII.2 and 24 (13.3%) as GII.P17_GII.17 (variant Kawasaki 2014) (Table 2).

**Table 2:** Distribution of pol and cap genotypes in 240 NoV strains typed in 2016. In grey tone the relevant pol/cap genotypes.

	GII.1	GII.2	GII.3	GII.4	GII.6	GII.7	GII.8	GII.13	GII.14	GII.15	GII.17	GII.20	GII.21	GI.3	GI.6	NT	TOT
GII.P2		18														1	19
GII.P4				33									1	1			35
GII.P7				1	7	1					1						10
GII.P8							2										2
GII.P15										1							1
GII.P16		35		7												2	44
GII.P17											24					2	26
GII.P21			3					2					1				6
GII.Pe				39													39
GI.P3														2	1		3
NT	1	26		14	2	1			1		8	1	1				55
TOT	1	79	3	94	9	2	2	2	1	1	33	1	3	3	1	5	240

**Fig 2:**
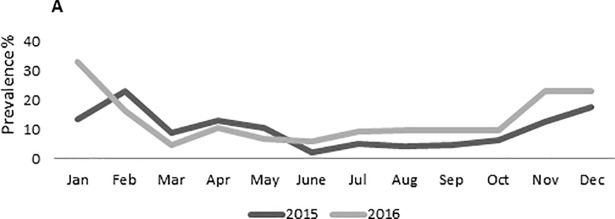
**Monthly prevalence (%) of NoVs in hospitalized pediatric population in Italy during 2015 (gray line) and 2016 (black line)**.

The GII.P16 strains were detected starting from April 2016 but only sporadically until October. In November-December, we registered a sharp increase in GII.P16 NoV prevalence, correlated with the seasonal increase in NoV activity (Fig 2). About 31.2% (98/315) of the 2016 NoV confirmed cases clustered in this period, with 80.0% (32/40) of the pol-typed strains being characterized as GII.P16 and only 12.5% (5/40) as GII.Pe. In the same time span, 63.5% (47/74) of the cap-typed strains were characterized as GII.2 and 28.4% (21/74) as GII.4. Overall, in 2016 the two recombinant pol/cap combinations, GII.P16_GII.2 and GII.P16_GII.4 accounted for 19.4% (35/180) and 3.9% (7/180) of the fully typed strains, respectively.

### Clinical features

Differences were found when analysing the demographical data of the patients with GII.2 infections versus cases attributed to GII.4 NoVs. The mean and median age of GII.2-infected patients was significantly (P<0.005) higher (5.7 and 4.6, respectively) than that of GII.4 cases (3 and 2 years, respectively).

## Discussion

A consistent circulation of non-GII.4 NoVs was observed for the first time in Italy during 2016. This apparent change in NoV epidemiology was mainly sustained by the emergence of novel recombinant GII.P16_GII.2 NoV strains. The simultaneous onset and spread worldwide of GII.2 and GII.4 recombinant viruses with the same GII.P16 pol in 2016 has posed questions concerning their origin. GII.P16 pol has been associated with seven different cap genotypes (GII.2, GII.3, GII.4, GII.10, GII.13, GII.16, and GII.17) [14]. GII.P16_GII.2 strains have been circulating since 2011, and since then, they have shown both in the pol and cap genes a linear evolution. This suggests that the GII.4 Sydney 2012 strain acquired the GII.P16 pol from co-circulating GII.P16_GII.2 strains [14]. Shortly after emerging in 2011, the GII.4 Sydney 2012 strain generated recombinant forms, with a GII.P4 pol, derived from the GII.P4_GII.4 pandemic variant New Orleans 2009, replacing the original GII.Pe pol [18]. In Italy, the recombinant GII.P4_GII.4 strain acquired a major epidemiological role in 2015–2016, accounting for 40.0% of the fully typed strains in 2015 and for 18.3% in 2016. Interestingly, in 2016 we monitored a third recombinant form of strain GII.4 Sydney 2012. This GII.4 recombinant has a GII.P16 pol and minimal changes in the cap compared to the previous GII.4 recombinants, and accounted for 3.8% of the strains fully typed in Italy 2016. Such a consistent circulation of GII.2 viruses had never been signaled before by NoV surveillance in Italy and it was mainly sustained by the novel recombinant GII.P16_GII.2 strain. Also, a marked circulation of GII.P2_GII.2 viruses (10.0%, 18/180) was monitored during our 2016 surveillance. This hospital-based surveillance study may not be representative of viruses circulating in the community, since ISGEV monitoring is restricted to severe hospitalised paediatric cases. However, a similar epidemiological change was observed in several studies focusing on environmental water, human outbreaks and sporadic cases from inpatients [8,9].

A tendency to infect older patients has been observed for non-GII.4 NoVs, such as GII.17 strains, when being newly introduced [10,23]. In our surveillance, some differences were found when we compared the demographical data of the patients with GII.2 infections with cases attributed to GII.4 NoVs. The mean and median age of GII.2 patients was significantly (P<0.005) higher (5.7 vs 3 and 4.6 vs 2 years, respectively) than that of GII.4. A similar age-related pattern has also been observed for GII.P16_GII.2 viruses in a recent study conducted in Hong Kong [24]. These observations seem to be consistent with a lack of herd immunity in the population, allowing the novel viruses to infect older patients more easily than GII.4 strains.

## Conclusions

ISGEV hospital-based surveillance monitored a marked increase in the circulation of the emerging GII.P16_GII.2 NoV strains in late 2016 (November-December). The spread of GII.P16 NoVs in Italy mirrors what has been depicted in several European countries in the same time span [12,13]. Moreover, on a year-to-year comparison, a 50% decrease of GII.4 viruses (60.9% vs 40.0% prevalence) and a nearly 4-fold increase of GII.17 NoVs (3.6 vs 14.0%) were observed (Tables 1 and 2). In recent years, novel non-GII.4 NoV strains (i.e. GII.2 and GII.17) have been observed as predominant genotypes in children hospitalized with acute gastroenteritis in several countries. NoV vaccines are in phase I and II clinical trials and they target either GI.1 or GII.4 or both NoV genotypes [25]. The global spread of non-GII.4 NoVs might pose a challenge for the development of NoV vaccines, as it is not clear whether, and to what extent, there is cross-protection between vaccine antigens and GII.2 and GII.17 viruses.
